# CT-to-MRI translation of medical volume data based on an enhanced diffusion model

**DOI:** 10.1038/s41598-026-45181-1

**Published:** 2026-03-23

**Authors:** Ji Ma, Jinjin Chen, Aoxiang Liang

**Affiliations:** 1 School of Ocean Information Engineering, Jimei University, Xiamen, China; 2 School of Design and Art, Communication University of Zhejiang, Hangzhou, China; 3 School of Computer Science and Technology, Zhejiang University of Technology, Hangzhou, China

**Keywords:** Biomedical engineering, Computer science, Information technology

## Abstract

In clinical practice, medical inter-modality imaging results can assist doctors in making better decisions, as different modalities imaging results can provide complementary information. Traditionally, obtaining these imaging results requires using various medical devices to scan patients, which can be time-consuming, costly, and potentially harmful to the patient. Motivated by the need to address these limitations, we propose an alternative method that facilitates the conversion of volume CT into volume MRI. The method is based on a Diffusion model and incorporates a post-processing approach to enhance the model’s output. To validate our approach, we conduct experiments and achieve good results on brain and pelvic datasets obtained from clinical practice, despite approximately 6% of the slices being incompletely paired. We also compare our method with state-of-the-art techniques, both qualitatively and quantitatively. Our experimental results show that our method outperforms state-of-the-art techniques, including MedSynthesisV1, CycleGAN, Pix2Pix and Diffusion, when using ground truth as a reference. Finally, we conduct an experiment to select the optimal hyperparameters, including the number of epochs and the parameters $cutoffPercentage\_left$ and $cutoffPercentage\_right$.

## Introduction

**Fig. 1:**
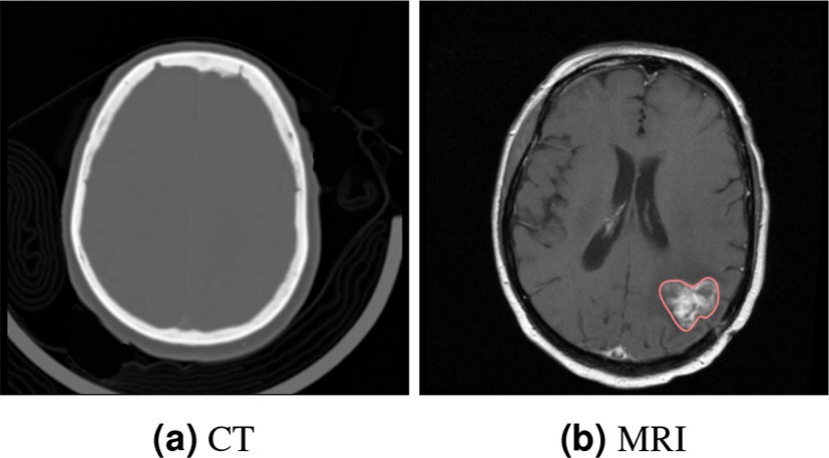
(**a**) CT and (**b**) MRI imaging results from the same patient.

Medical inter-modality imaging results are crucial for the diagnosis and treatment of various diseases, as different imaging modalities provide distinct and essential types of information. For example, CT (Computed Tomography) offers high-resolution anatomical details, especially for bones, lungs, and hemorrhages, making it particularly suitable for trauma assessment (such as fractures and internal bleeding), pulmonary diseases, and tumor detection. CBCT (Cone Beam Computed Tomography), while similar to CT, is better suited for imaging small regions such as the head, maxillofacial area, and teeth. It is primarily used in dental implant planning, evaluation of jawbone structure, and ENT (ear, nose, and throat) imaging. In contrast, MRI (Magnetic Resonance Imaging) provides clear visualization of soft tissue structures such as the brain, spinal cord, joints, muscles, and internal organs. It is mainly used for diagnosing neurological conditions (such as brain tumors and strokes), spinal disorders, joint injuries, and abdominal or pelvic tumors. PET (Positron Emission Tomography), on the other hand, provides metabolic and functional information, enabling the observation of tissue or organ metabolic activity. It is commonly used in cancer detection and staging, the assessment of brain metabolic disorders (e.g., Alzheimer’s disease), and the diagnosis of cardiovascular diseases. Each imaging modality focuses on different aspects of the human body, complementing one another’s limitations. This integration enables physicians to form more complete and accurate diagnoses. Furthermore, different modalities may reflect the same pathological condition through different types of information, thereby providing additional evidence to support diagnosis. Therefore, combining multiple imaging modalities can help clinicians make more informed decisions, supporting disease screening, diagnosis, and image-guided therapy. As illustrated in Fig. 1, the CT and MRI scans of the same patient are presented. In the CT scan, the skull is clearly visible, but the soft tissue structures are difficult to distinguish, making it challenging to identify abnormalities in the brain. In contrast, the MRI scan clearly displays the brain’s soft tissue anatomy, and the red highlighted region shows a lesion more prominently, which may indicate a brain tumor. Compared to CT, MRI provides different and valuable information, offering physicians additional support in identifying lesions and tumors. However, obtaining these inter-modality imaging results traditionally requires multiple scans using different devices, which is not only costly and time-consuming but may also pose risks to the patient. For example, in emergency situations—such as when a patient’s condition is critical and there is no time or feasibility to perform an MRI scan—it becomes necessary to generate MRI images from existing CT scans to support clinical diagnosis. Moreover, MRI machines are expensive, and most general hospitals typically have only one unit. Excessive scanning time can delay treatment and hinder effective clinical intervention. In such scenarios, generating MRI from CT can save significant time and provide timely support for diagnosis and treatment planning.

With advancements in deep learning and artificial intelligence technologies, many medical image conversion methods have been developed to reduce reliance on multiple imaging devices and generate high-quality inter-modality data, such as MRI-to-CT^1–4^, CT-to-MRI^5,6^, PET-to-CT^7^, CBCT-to-CT^8,9^. These methods all utilize similar training and inference processes. During training, the medical imaging result from one modality is used as input to the model, while another modality is used as output, allowing the model to learn the mapping relationship between the two. During inference, this mapping relationship is used to convert medical imaging results from one modality into another. Moreover, these types of methods has undergone clinical validation, proving its effectiveness and is already in use in various fields^10^. However, most of the implemented methods focus on image-to-image medical inter-modality translation, with relatively few studies addressing volume-to-volume medical inter-modality translation. And in the medical field, most imaging results are in the form of volumetric data. If we only perform image-to-image translation, we risk losing significant spatial information, which can be detrimental to 3D reconstruction and visualization analysis. However, compared to image-to-image medical inter-modality translation, volume-to-volume translation is a more challenging task. There are two core reasons: First, volume-to-volume translation requires volumetric data, which are larger in size compared to image data. This results in higher acquisition costs and smaller volumetric data, potentially impacting the effectiveness of model training. Additionally, some tasks require data annotation, and annotating volumetric data involves labeling every slice, which is more challenging and time-consuming. Secondly, to continually improve the performance of volume-to-volume translation models, it is essential to consider the relationships between each slice in the volumetric data, a factor that is not relevant for image data. Besides, except for the challenges associated with transitioning from image data to volumetric data, we also need to consider the specific challenges inherent to medical imaging. In fact, collecting paired medical images involves scanning the same regions at two different time points. Therefore, the imaging results are likely to be affected by operational differences and short-term variations, leading to incomplete pairing between different modalities.

### Key contributions

To tackle the aforementioned challenges in volume-to-volume medical image translation, especially under unpaired and limited data scenarios, this paper makes the following key contributions: (1) A Novel Unified Framework for Unpaired Volumetric Translation. We propose a novel framework that integrates a Diffusion Model with a tailored post-processing procedure. This framework is specifically designed for volumetric CT-to-MRI translation and is robust to incompletely paired or unpaired datasets, addressing a critical practical bottleneck in clinical data collection. (2) We introduce enhanced-DDPM, whose novelty is underpinned by two custom-designed neural modules: Transform Block (TB). A novel dual-branch 3D residual block that promotes richer multi-scale feature extraction and more stable gradient propagation during the training of deep volumetric networks, leading to enhanced detail preservation. Self-Attention Less Memory Block (SALMB). A breakthrough memory-efficient attention mechanism for 3D diffusion. Unlike standard self-attention, which is prohibitively expensive for volumetric data, SALMB intelligently integrates average pooling and upsampling operations. This unique design retains the ability to model long-range voxel dependencies—crucial for anatomical consistency—while reducing memory consumption, enabling high-resolution volumetric diffusion models to run feasibly on standard hardware. (3) Comprehensive Experimental Validation and Ablation Studies: We conduct extensive experiments to validate our approach. This includes both qualitative and quantitative comparisons against several state-of-the-art models (e.g., MedSynthesisV1, CycleGAN, Pix2Pix, and standard Diffusion models). Furthermore, we perform detailed ablation studies to demonstrate the effectiveness of each component in our framework and to identify the optimal hyperparameters for the task.

## Related works

We classify medical inter-modality translation methods into three types based on the models used: GAN-based, Transformer-based, and Diffusion-based medical inter-modality translation. In the past decade, numerous studies have proposed using various deep learning models to achieve it. We recommend readers refer to a review on medical inter-modality translation by McNaughton et al.^11^ This review covers various deep learning methods, including GANs and CNNs, used to generate medical imaging results across different modalities such as MRI and CT.

### GAN-based inter-modality medical image translation

For GAN-based inter-modality medical image translation, Patashnik et al.^12^proposed an innovative GAN framework to address imbalances between source and target domains in cross-modal image translation, enhancing translation performance through cross-modal transfer and adversarial mechanisms. Huang et al.^13^ presented a multi-constraint transferable GAN approach that significantly enhances the quality and consistency of cross-modal brain image synthesis. Gu et al.^1^ innovatively enhanced MRI-to-CT image translation by using a multi-generative network and perceptual supervision, resulting in more detailed and accurate synthesized images. Ranjan et al.^2^ introduced a GAN-based approach for synthesizing CT images from T2-weighted MRI data, improving image registration and dose planning in MR-guided radiation therapy. Jin et al.^14^ introduced a semi-supervised learning approach that leverages generative adversarial networks to convert CT images into MR images, improving quality and reducing reliance on labeled data. Lee et al.^15^ provided an effective solution for cross-modal synthesis of spinal images using GAN technology. Xu et al.^5^ introduced a bidirectional GAN framework that leverages multiple generators and adversarial networks, combined with spectral normalization and localization techniques, to effectively perform CT-to-MRI image translation and vice versa. This approach enhanced image quality while preserving structural consistency and detail. Li et al.^6^ presented a deep learning method to convert brain CT images into high-quality MRI images, providing an effective solution for MR-guided radiotherapy. Kida et al.^16^ improved the visual quality of CBCT images by using a CycleGAN framework to transform low-quality CBCT images into high-quality CT-like images, enhancing their visualization. Armanious et al.^17^ presented a GAN-based deep learning method for effective attenuation correction of whole-body [18F]FDG-PET images without relying on auxiliary CT scans, enhancing the quantitative accuracy of the PET images. Liu et al.^18^ introduced a deep learning-based attenuation correction method that uses CNNs to generate accurate PET attenuation maps from CT images, improving PET imaging accuracy and diagnostic quality. Touati et al.^3^ introduced FIGAN, which improves the synthesis of head and neck MRI and CT images by preserving feature consistency, achieving higher-quality and more accurate synthetic images.

### Transformer-based inter-modality medical image translation

For Transformer-based inter-modality medical image translation, Chen et al.^19^ introduced a cross-modal prompting mechanism that enables effective multi-task learning in medical image translation, improving performance and consistency across different tasks. Dai et al.^20^ introduced a Transformer-based approach to advance cross-modal medical image translation by using self-attention mechanisms to better capture inter-modality relationships, leading to improved translation performance. Yan et al.^21^ proposed a Swin Transformer-based GAN architecture that leverages self-attention mechanisms to improve the quality and detail retention in multi-modal medical image translation. Zhao et al.^4^ presented a method that integrates residual networks and transformers within a GAN framework, improving the accuracy and detail of CT images synthesized from MR images. Hu et al.^22^ leveraged the Transformer architecture to enhance information exchange between different medical image modalities, improving synthesis accuracy and quality.

### Diffusion-based inter-modality medical image translation

For diffusiion-based inter-modality medical image translation, Zhu et al.^23^introduced a novel approach based on latent Diffusion models for high-quality cross-modality 3D brain MRI synthesis and implemented an effective latent space modeling technique that generates realistic 3D brain images across different modalities while preserving structural and textural details. Wang et al.^24^ presented a zero-shot Diffusion model method for cross-modality medical image translation and segmentation, achieving effective modality mapping without paired images. Kim et al.^25^ proposed an Adaptive Latent Diffusion Model that improves the precision and efficiency of 3D medical image translation by adaptively adjusting the Diffusion process in latent space. Wang et al.^26^ demonstrated the potential of Diffusion models for cross-modal image translation, excelling in the details and diversity of the generated images. Özbey et al.^27^ introduced a method using adversarial Diffusion models for unsupervised medical image translation. This approach leveraged the adversarial mechanism of Diffusion models to achieve high-quality cross-modal image synthesis without paired training data, significantly improving the realism and consistency of the generated images. The aforementioned diffusion-based methods have demonstrated the powerful potential of generative models for cross-modal synthesis. Notably, Zhu et al.^23^ and Kim et al.^25^ have pioneered the extension of diffusion models to 3D volumetric medical image translation, setting important benchmarks. However, these pioneering 3D approaches often face two intertwined practical challenges: (1) a heavy reliance on perfectly paired training data, which is difficult to acquire in clinical practice, and (2) the prohibitive computational and memory overhead associated with applying standard transformer-based self-attention mechanisms to high-resolution 3D volumes, limiting their feasibility in resource-constrained environments.

### Others

For other methods, Zhu et al.^28^ introduced a method for cross-domain medical image translation using a shared latent Gaussian mixture model, and this approach enhances the quality and consistency of generated images by effectively managing image translation between different modalities while preserving structural and semantic coherence. Thummerer et al.^9^ compared different CBCT-based synthetic CT methods to improve proton dose calculations and accuracy in adaptive proton therapy.

As mentioned above, most of the existing works focus on image-based inter-modality translation, with few studies addressing volumetric inter-modality translation, which is very useful for 3D reconstruction and visualization. Therefore, in this paper, we focus on volumetric inter-modality translation.

## Framework overview

**Fig. 2:**
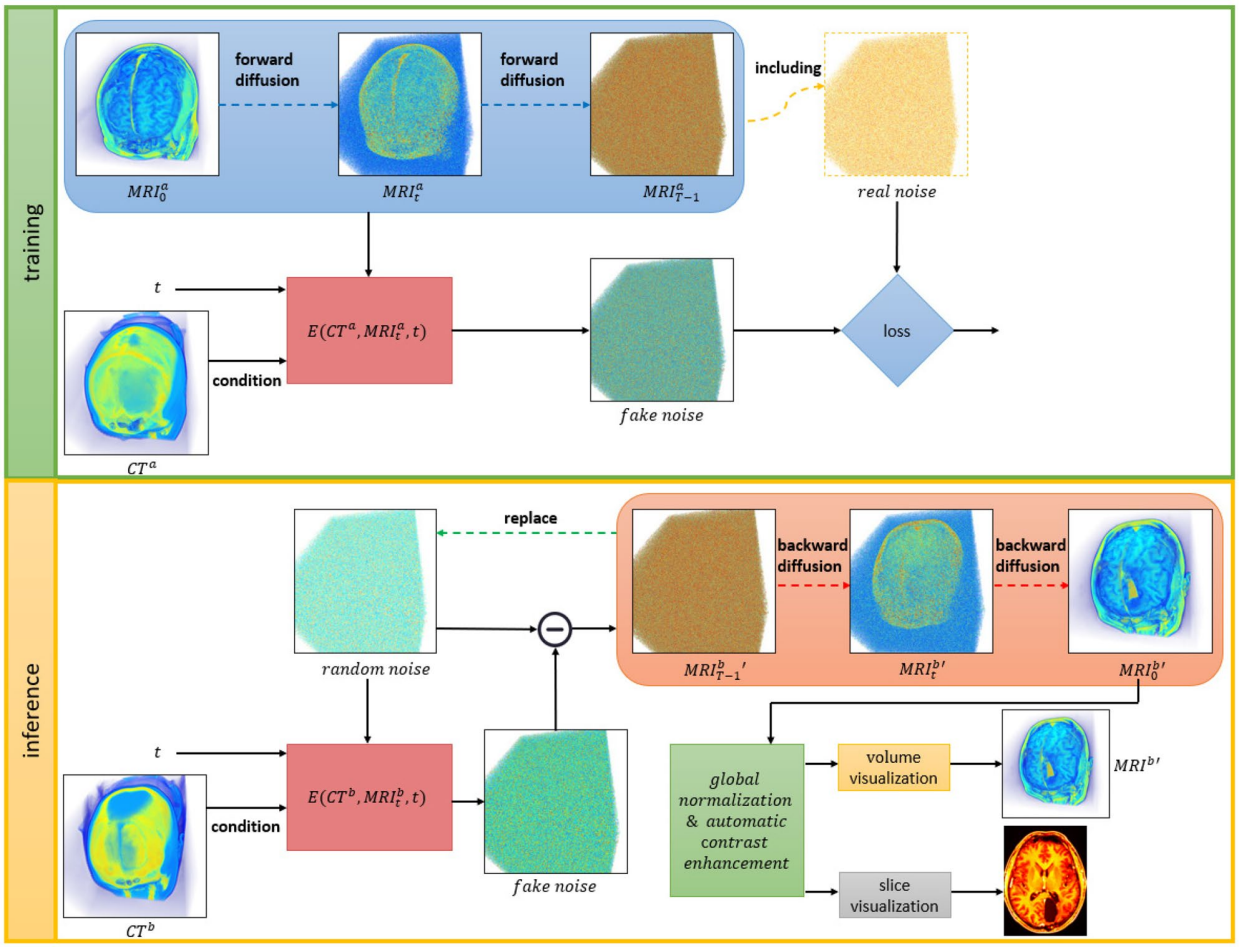
Research framework.

The framework of our research is depicted in Fig. 2. Before introducing this framework, we first define the symbols used throughout. Our method aims to generate volume MRI from volume CT, which we denote as *MRI* and *CT*, respectively. Since our method is based on a Diffusion model, we use *t* to denote the time step, and $MRI_{t}$ to represent volume MRI at time step *t*. $MRI_{0}$ denotes the volume MRI at time step 0, which represents the original volume MRI, and $MRI_{T-1}$ denotes the volume MRI at the final time step, where *T* represents the total number of time steps. To distinguish, we respectively use *a* and *b* to denote them during the training phase and the inference phase. We use a prime (’) to denote volumetric data generated by the model. Finally, since our network is named enhanced-DDPM, we use *E* to represent the mapping function.

The blue rounded rectangular box in Fig. 2 represents the forward Diffusion process. Its function is taking the original data $MRI_{0}^{a}$, the random time step *t* and the random noise as inputs and generating the data $MRI_{t}^{a}$ by adding the noise to the data. The orange rounded rectangular box in Fig. 2 represents the backward Diffusion process, which has the opposite function of the forward Diffusion process. It takes the data $MRI_{t}^{b}$’, the corresponding time step *t*, and the associated noise as input, and outputs the data $MRI_{t-1}^{b}$’ by recovering the original data from the noisy version.

The green box in the Fig. 2 represents the training phase. Given paired $CT^{a}$ and $MRI_{0}^{a}$, we first input $MRI_{0}^{a}$ and a random time step *t* into the forward Diffusion process to obtain $MRI_{t}^{a}$. Besides, in each forward Diffusion process, there is a corresponding real noise that is used to compute the loss at the end of the training phase. Then, we input $MRI_{t}^{a}$ and the corresponding time step *t* into our model, using the corresponding $CT^{a}$ as a condition to generate a fake noise. Finally, we compare it with the real noise as a loss function to allow the model to learn the relationship between CT and MRI.

The yellow box in Fig. 2 illustrates the reference phase, which aims to generate the corresponding $MRI_{0}^{b}$’ given $CT^{b}$. Firstly, we use random noise $MRI_{T-1}^{b}$’ as the start for the reverse Diffusion process. We feed $MRI_{T-1}^{b}$’ and the time step $T-1$ into our model, using $CT^{b}$ as a condition, and obtain a predicted noise. We then input $MRI_{T-1}^{b}$’, the time step $T-1$, and the predicted noise into the backward Diffusion process, resulting in $MRI_{T-2}^{b}$’. At this point, we replace $MRI_{T-1}^{b}$’ with $MRI_{T-2}^{b}$’ and repeat the above process. This iterative process continues until we obtain $MRI_{0}^{b}$’, which is our goal. Then, we apply global normalization and automatic contrast enhancement to improve the quality of the results. Finally, we conduct volume and slice visualizations to compare with other methods.

## Enhanced-DDPM

### Diffusion process

Our model is built upon the Denoising Diffusion Probabilistic Models (DDPM) framework. The core idea is to define a forward process that systematically corrupts training data with noise, and then learn a reverse process that inverts this corruption to generate data from noise. Forward process: Let $\textbf{x}_0 \sim q(\textbf{x}_0)$ represent a ground-truth volumetric MRI scan. The forward process is a fixed Markov chain that gradually adds Gaussian noise over $T$ timesteps, according to a variance schedule $\beta _1, \dots , \beta _T$:

$$q(\textbf{x}_{1:T} | \textbf{x}_0) = \prod _{t=1}^{T} q(\textbf{x}_t | \textbf{x}_{t-1}), \quad \text {where} \quad q(\textbf{x}_t | \textbf{x}_{t-1}) = \mathcal {N}(\textbf{x}_t; \sqrt{1-\beta _t} \textbf{x}_{t-1}, \beta _t \textbf{I}).$$

A notable property is that we can directly sample $\textbf{x}_t$ at an arbitrary timestep $t$ in closed form:

1 $$\begin{aligned} \textbf{x}_t = \sqrt{\bar{\alpha }_t} \textbf{x}_0 + \sqrt{1-\bar{\alpha }_t} \boldsymbol{\epsilon }, \quad \boldsymbol{\epsilon } \sim \mathcal {N}(\textbf{0}, \textbf{I}), \end{aligned}$$

where $$\alpha _t = 1 - \beta _t$$ and $$\bar{\alpha }_t = \prod _{s=1}^{t} \alpha _s$$.

Reverse process: The generative (reverse) process is defined as another Markov chain parameterized by our network $E$. Starting from pure noise $\textbf{x}_T \sim \mathcal {N}(\textbf{0}, \textbf{I})$, it is defined as:

$$p_\theta (\textbf{x}_{0:T}) = p(\textbf{x}_T) \prod _{t=1}^{T} p_\theta (\textbf{x}_{t-1} | \textbf{x}_t), \quad \text {where} \quad p_\theta (\textbf{x}_{t-1} | \textbf{x}_t) = \mathcal {N}(\textbf{x}_{t-1}; \boldsymbol{\mu }_\theta (\textbf{x}_t, t), \sigma _t^2 \textbf{I}).$$

The mean $\boldsymbol{\mu }_\theta (\textbf{x}_t, t)$ is predicted by the network. Following the standard simplification in, we reparameterize the network to directly predict the noise component $\boldsymbol{\epsilon }$. Given the conditional input CT volume $\textbf{c}$, our network $E$ takes the concatenated noisy volume $[\textbf{x}_t, \textbf{c}]$ and the timestep $t$, and outputs the predicted noise:

2 $$\begin{aligned} \boldsymbol{\epsilon }_\theta = E([\textbf{x}_t, \textbf{c}], t). \end{aligned}$$

Training objective: The model is trained to maximize the variational lower bound on the likelihood. As derived in , this objective simplifies remarkably to a noise prediction task. For a training pair $(\textbf{x}_0, \textbf{c})$, we uniformly sample a timestep $t \sim \mathcal {U}(\{1, \dots , T\})$ and noise $\boldsymbol{\epsilon } \sim \mathcal {N}(\textbf{0}, \textbf{I})$, and compute the loss:

3 $$\begin{aligned} \mathcal {L} = \mathbb {E}_{\textbf{x}_0, \textbf{c}, \boldsymbol{\epsilon }, t} \big [ \Vert \boldsymbol{\epsilon } - E([\textbf{x}_t, \textbf{c}], t) \Vert _1 \big ]. \end{aligned}$$

### Network architecture

**Fig. 3:**
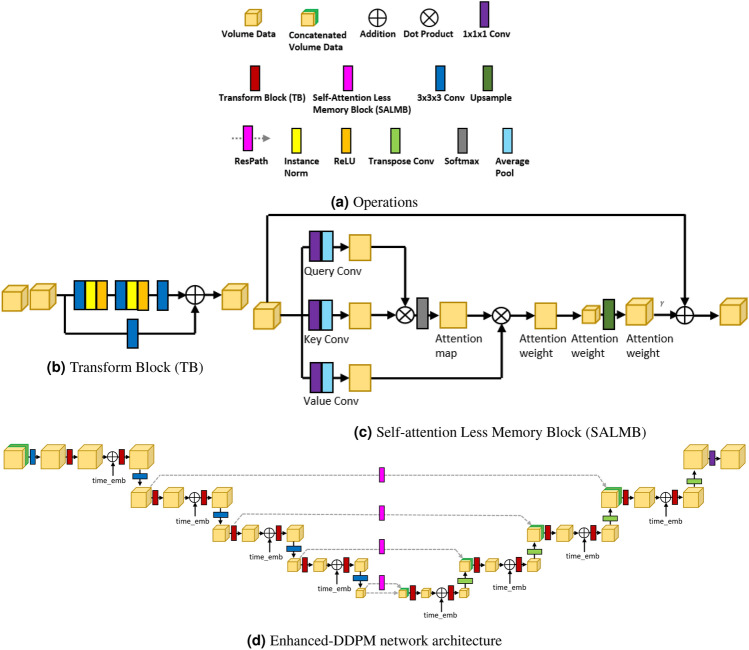
(**a**) Operations, (**b**) Transform Block (TB for short), and (**c**) Self-attention Less Memory Block (SALMB for short) used in the Enhanced-DDMP network architecture. (**d**) The network architecture of Enhanced-DDPM.

Figure 3 shows the network architecture of our model, designed to transform the noisy volume MRI and the corresponding time step into the noise used during the forward Diffusion process, while using the volume CT as a condition to guide the network’s learning. Due to the challenges in acquiring paired volume CT and MRI, as well as the potential for imperfect pairing at the slice level, our model must not only learn the relationships within a few paired volumetric data but also demonstrate robustness to unpaired data. And before introducing the network architecture, we define the basic operations in Fig. 3a, which will be used in network architecture.

The overall architecture of the network, as shown in the Fig. 3d, is based on the UNet network with modifications and innovations. First, to achieve the goal of converting volume CT to volume MRI, we choose to use the volume CT as a condition to guide the transformation. Saharia et al.^29^ pointed out that during the use of Diffusion models, conditions can be used for model training. The condition and the original data can be concatenated and fed into the model for training, which can yield better results. Then our network architecture follows an encoder-decoder structure, where the encoder’s task is to extract features from large datasets, and the decoder’s task is to decode these features and expand them into outputs. At each layer, they should have matching scale factors for proper upsampling and downsampling. Specifically, it involves four encoding and four decoding layers. During each encoding layer, two transform blocks are used, and in the second transform block, the time step embedding $time\_emb$, which is derived from the time step through certain transformations, is added. Then it is followed by a 3D convolution operation for downsampling. In each decoding layer, two transform blocks are also used, which are similar to those in the encoding layer. The difference is that in the decoding layers, they use deconvolution for upsampling, which is the reverse of the encoding process. The encoding and decoding layers are connected not only through the bottom-most module but also via the ResPath in the intermediate layers. Shallow layers capture detailed information from the data, while deeper layers capture overall structure and more complex patterns. Passing both types of information helps not only to restore details but also to provide a global semantic understanding.

Besides, the core of our UNet primarily relies on the Transform Block (TB) and the Self-attention Less Memory Block (SALMB). As shown in the Fig. 3b,c , their detailed internal structures are illustrated. A TB is essentially a residual block. Upon entering this module, the network path splits into two branches. One branch passes through a simple 3D convolution, while the other passes through two combination of a 3D convolution, a normalization layer, and an activation function, along with one additional 3D convolution. The results from both branches are then added together. The main advantages of this module are that it can extract richer and more detailed features during forward propagation and facilitate gradient propagation during backward propagation. SLMB is an improvement based on the self-attention module. Similar to the self-attention module, it first uses query convolution, key convolution, and value convolution to obtain the query, key, and value. The self-attention module typically proceeds directly with the softmax and the attention map. However, our module first compresses the data using average pooling before applying the softmax and generating the attention map. And to ensure that the output data matches the input data in terms of volume, we perform upsampling after converting the data back to the volumetric format. Additionally, we include a residual connection at the end. The reasons for adopting this approach are: First, unlike convolutions, the self-attention module enables the model to learn from distant voxels, which enhances the accuracy of the results. Second, by incorporating average pooling and upsampling, our method is more storage-efficient compared to traditional self-attention mechanisms.

### Loss functions

4 $$\begin{aligned} L^{E} =\left\| real\ noise_{MRI_{t} } - E\left( CT, MRI_{t}, t \right) \right\| _{L1} \end{aligned}$$

Since the overall architecture of our method is based on Diffusion models, our loss function is the same as that used in Diffusion models, as shown in the Eq. (1). Here, *E* represents our network, $real noise_{MRI_{t}}$ denotes the true noise that is added to MRI data at time step *t*, and *E*() denotes the network’s output, so our loss is the L1 norm between the real noise and the network’s output. Using this loss function allows the network to identify more accurate noise from the noisy data, thereby enabling the backward Diffusion process to denoise the data and recover the target results.

## Global normalization and automatic contrast enhancement

**Algorithm 1:**
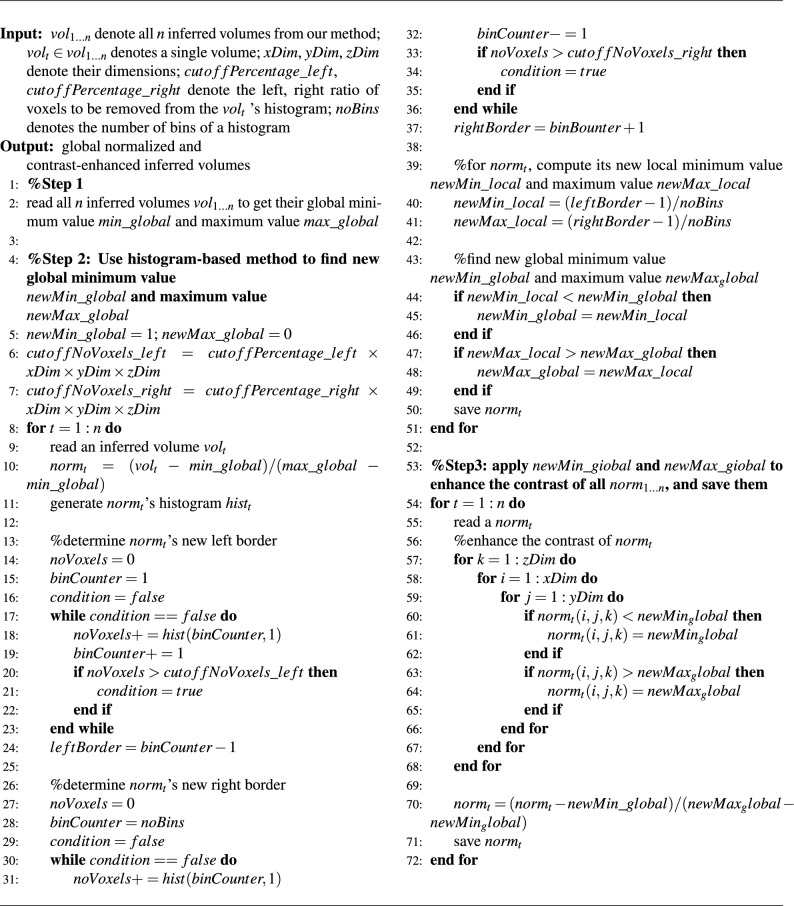
global normalization & automatic contrast enhancement

In medical image analysis, intensity normalization is a critical preprocessing step that reduces variability across scanners and acquisition protocols, thereby improving the robustness of deep learning models. Common approaches, such as global histogram-based normalization, primarily aim to map intensity distributions of different images into a common range. While effective for achieving global consistency, these methods do not necessarily enhance local contrast, which is important for both human interpretation and model discriminability. To address this, we employ a two-stage post-processing pipeline: first, a global intensity normalization is applied to align all volumes to a standardized intensity scale; second, an automatic contrast enhancement (ACE) step adaptively adjusts local contrast to make anatomical boundaries and potential lesions more distinct. This combined strategy not only ensures cross-sample comparability but also improves intra-image clarity, facilitating visual inspection and potentially providing more discriminative input features for subsequent automated analysis. The importance of tailored preprocessing in medical imaging is also supported by recent literature—for instance, Umirzakova et al.^30^ highlighted that careful image normalization and enhancement contribute significantly to the performance of MRI-based lesion classification tasks.

Before training the model, we preprocess the dataset by normalizing it to a range of 0 to 1. However, our method is based on Diffusion model, which adds Gaussian noise to the original data. Since the Gaussian distribution is not limited to the range of 0 to 1, the range of the results generated through the backward Diffusion process, where the model uses Gaussian noise for training, may also exceed this range, which is inconsistent with the original data range. This discrepancy can lead to poor results when analyzing metrics. Therefore, we employ a novel post-processing algorithm to enhance the inference results. Our post-processing algorithm can be summarized as global normalization and automatic contrast enhancement. It takes as input all the results obtained from our model $vol_{1 \ldots n}$, the dimensions of the original data *xDim*, *yDim* and *zDim*, two parameters $cutoffPercentage\_left$ and $cutoffPercentage\_right$, and the number of histogram bins *nobins*. The output is the results after global normalization and contrast enhancement.

Our algorithm is presented in Algorithm 1. This algorithm consists of three main steps. In the first step, read all the inferred results to determine their global maximum value $max\_global$ and global minimum value $min\_global$. The second step focuses on contrast enhancement. Initially, use *xDim*, *yDim*, *zDim*, $cutoffPercentage\_left$ and $cutoffPercentage\_right$ to determine the number of voxels to be removed from both sides of the histogram. Next, sequentially read $vol_{1 \ldots n}$, perform local normalization, and generate its histogram. Then, use the determined number of voxels to be removed from both sides of the histogram to identify the minimum bins on the left side *leftBorder* and maximum bins on the right side *rightBorder*. Specifically, this involves iteratively removing voxels from both sides until the specified target is reached. Then, based on this adjusted histogram, determine the new local minimum values $newMin\_local$ and the local maximum values $newMax\_local$. Finally, compare each $newMin\_local$ and $newMax\_local$ with $min\_global$ and $max\_global$ to generate a final $min\_global$ and $max\_global$. The final step is global normalization using $min\_global$ and $max\_global$. Specifically, modify any voxel greater than $max\_global$ to match $max\_global$, and any voxel smaller than $min\_global$ to match $min\_global$. Finally, normalize the data to a range of 0 to 1 using $min\_global$ and $max\_global$.

## Visualization

5 $$\begin{aligned} O\left( v \right) =\begin{Bmatrix} inten\left( v \right) \times \gamma&inten\left( v \right) \in foreground\\ 0&otherwise\end{Bmatrix} \end{aligned}$$

To better visualize the differences between the results inferred by our method and other methods compared to the reference ground truth, we use both volume and slice visualizations to process all the inferred and reference data. For volume visualization, we map all inferred volumes from different methods to the ‘jet’ colormap. Furthermore, we use Eq. (2) to determine the opacity of each voxel of those inferred volumes. More specifically, *O*(*v*) denotes the opacity of each voxel *v*; *inten*(*v*) denotes the intensity of *v*; *y* denotes a user-specified parameter that is used to control the overall opacity of the visualization; foreground denotes the foreground of the volumes, which is defined within a range. For slice visualization, we map the inferred slices to the ‘hot’ colormap.

## Implementation

Our model is implemented using PyTorch and our device for training and inference is a remote Dell server with two NVIDIA GTX 3090 GPUs. For visualization, we use a local desktop with an NVIDIA GTX 1060 GPU. And for dataset, we follow the rule of thumb in machine learning^31^ that uses a ratio of 70%:30% of the total data for model training and inference. The total time step *T* used for forward and backward Diffusion in our models is 1000. The start and end values used to linearly compute the schedule of $\beta$ in the closed form are 0.0001 and 0.02, respectively. During training, for the sake of data augmentation, we crop the original volumes at random positions to be half of their dimensions in each x, y, z direction. The batch size, dimension of time embedding, hidden channels used in our model are 1, 64, and 32, respectively. The learning rate used for our model training is 0.00002. The *nobins* used in the global normalization and automatic contrast enhancement algorithm is 100. The $cutoffPercentage\_left$ and $cutoffPercentage\_right$ used in the global normalization and automatic contrast enhancement algorithm are 0.55 and 0.015 for the brain dataset, and are 0.48 and 0.05 for the pelvis dataset. The $\gamma$ used in the volume visualization is 0.3 for the brain dataset, and is 10 for the pelvis dataset. All the above-mentioned parameter values are determined experimentally. The readers can access the source code of the implementation in Github via https://github.com/mjslamdunk/EnhancedDiffusion/.

## Results and discussion

This section presents and analyzes the results of our experiments. In our experiments, we employ four additional methods for comparison to validate our method. The first technique, MedSynthesisV1, is a model designed for medical image translation tasks that extends the approach proposed by Nie et al.^32^ to handle both inter-modality and intra-modality image-to-image translation. The second technique is CycleGAN^33^, a type of generative adversarial network used for unpaired image-to-image translation tasks. The third technique is Pix2Pix^34^, another popular generative adversarial network used for paired image-to-image translation tasks. The fourth technique is Diffusion^35^, a class of generative models that generate data by gradually denoising a random noise sample. However, it can also be used for unpaired image-to-image translation tasks. Since our experiments are conducted on volumetric data, we adapt all the techniques to work with volumetric data. To compare their performance, we use two medical volumetric datasets and conduct relevant quantitative and qualitative experiments based on ground truth. Additionally, to validate our method, we conduct further experiments, including performance analysis, statistics of imperfectly-paired data, and parameter selection.

### Dataset description

**Table 1:** The dataset names, their dimensions, their data type, their total volumes, training volumes, and inference volumes used in the research.

Dataset	Dimension used in the research ($x \times y \times z$)	Data type	Total volumes	Training volumes	Inference volumes
Brain	$168 \times 224 \times 160$	$32-bit$ float	100	70	30
Pelvis	$384 \times 240 \times 96$	$32-bit$ float	100	70	30

The dataset we used is SynthRAD2023, a challenge introduced at the MICCAI 2023 conference, focused on synthesizing computed tomography images for radiotherapy. The dataset includes paired volumetric CT and MRI data for patients who have undergone radiotherapy, specifically in the brain or pelvis regions. The dataset was collected from three Dutch university medical centers and reflects typical clinical imaging protocols and scanners used in those centers. The MRI volumes in the SynthRAD 2023 dataset are T1-weighted (T1w) images, acquired using a standardized 3D T1-weighted gradient-echo sequence. As per the dataset description, the key acquisition parameters are: repetition time (TR) = 2300 ms, echo time (TE) = 2.98 ms, inversion time (TI) = 900 ms, flip angle = 9°, with an isotropic voxel size of 1.0 mm^3^. The use of a consistent acquisition protocol across all subjects ensures reproducibility for training but also implies that the trained model is specifically adapted to synthesize images with T1w-like contrast. For a detailed description of the dataset, please refer to Thummerer et al.^31^

In our experiments, Due to the substantial computational time required for training, a consecutive subset of the first 100 paired samples from the SynthRAD 2023 training set was used for both the brain and pelvis tasks. This subset was verified to be representative of the full dataset’s variability. To simplify processing and conserve storage space, we preprocess the dataset to specific dimensions. The dimensions of the processed data are shown in the Table 1.

### Qualitative analysis

**Fig. 4:**
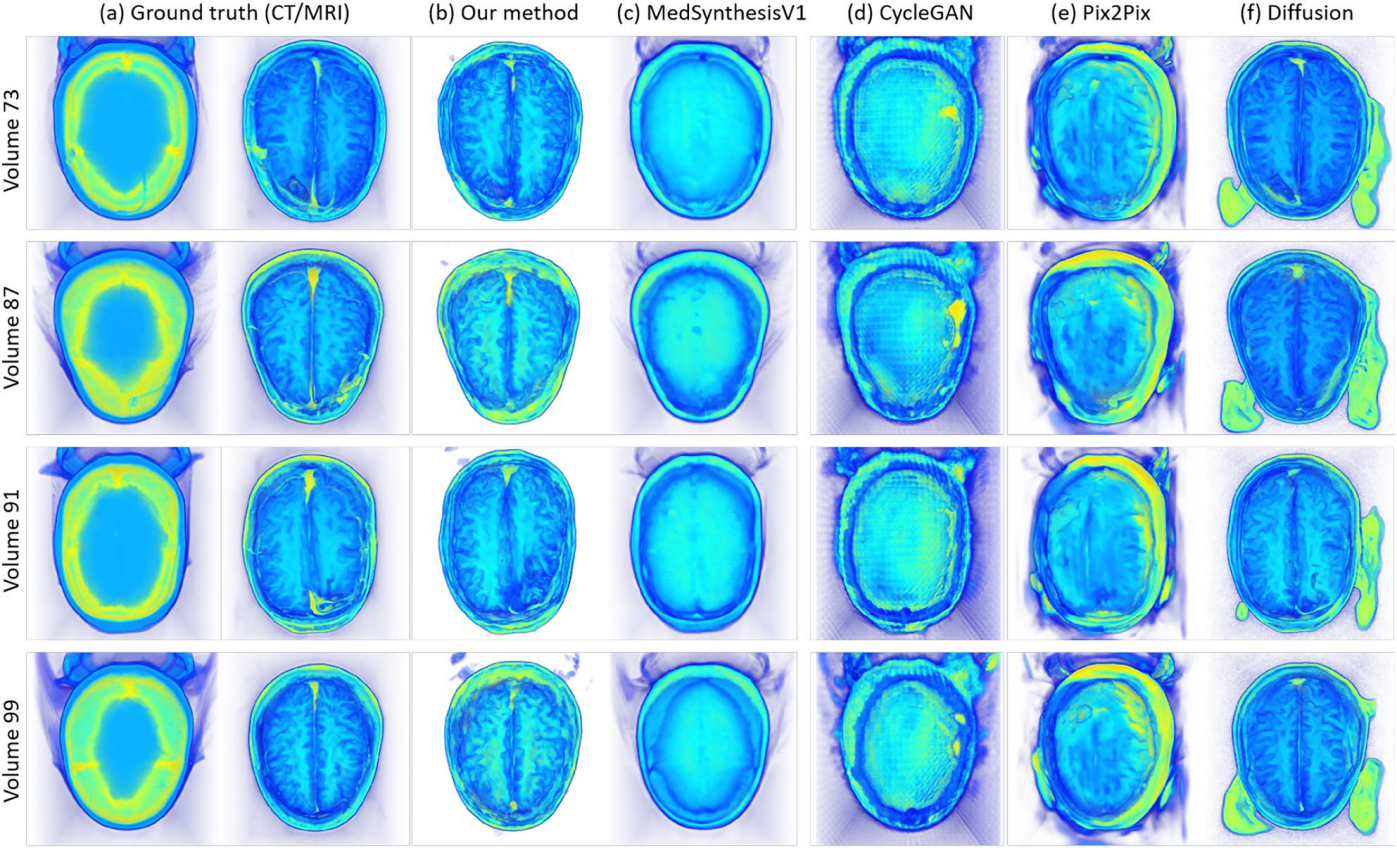
(**a**) volume visualization of the CT (left) and MRI (right) volumes from the ground truth, and (**b**–**f**) volume visualization of the synthesized MRI volumes from our method, MedSynthesisV1, CycleGAN, Pix2Pix, and Diffusion for the brain dataset. Each row denotes a specific volume with an associated number (the four volumes are randomly selected), and each column denotes a specific method used to generate those volumes.

**Fig. 5:**
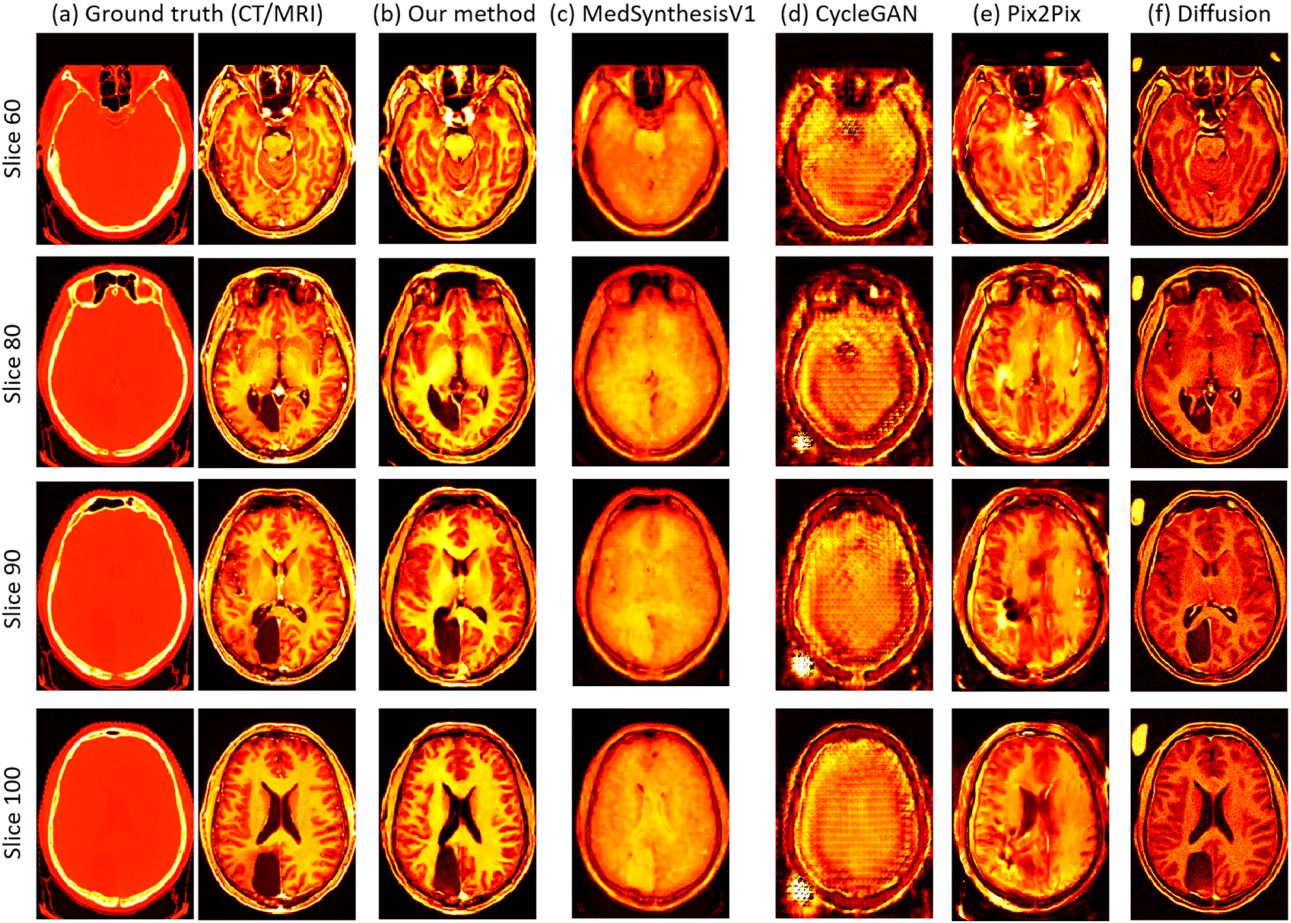
(**a**) slice visualization of the CT (left) and MRI (right) slices from the ground truth, and (**b**–**f**) slice visualization of the synthesized MRI slices from our method, MedSynthesisV1, CycleGAN, Pix2Pix, and Diffusion for the brain dataset. Each row denotes a specific slice with an associated number (the four slices are randomly selected from volume 71), and each column denotes a specific method used to generate those slices.

**Fig. 6:**
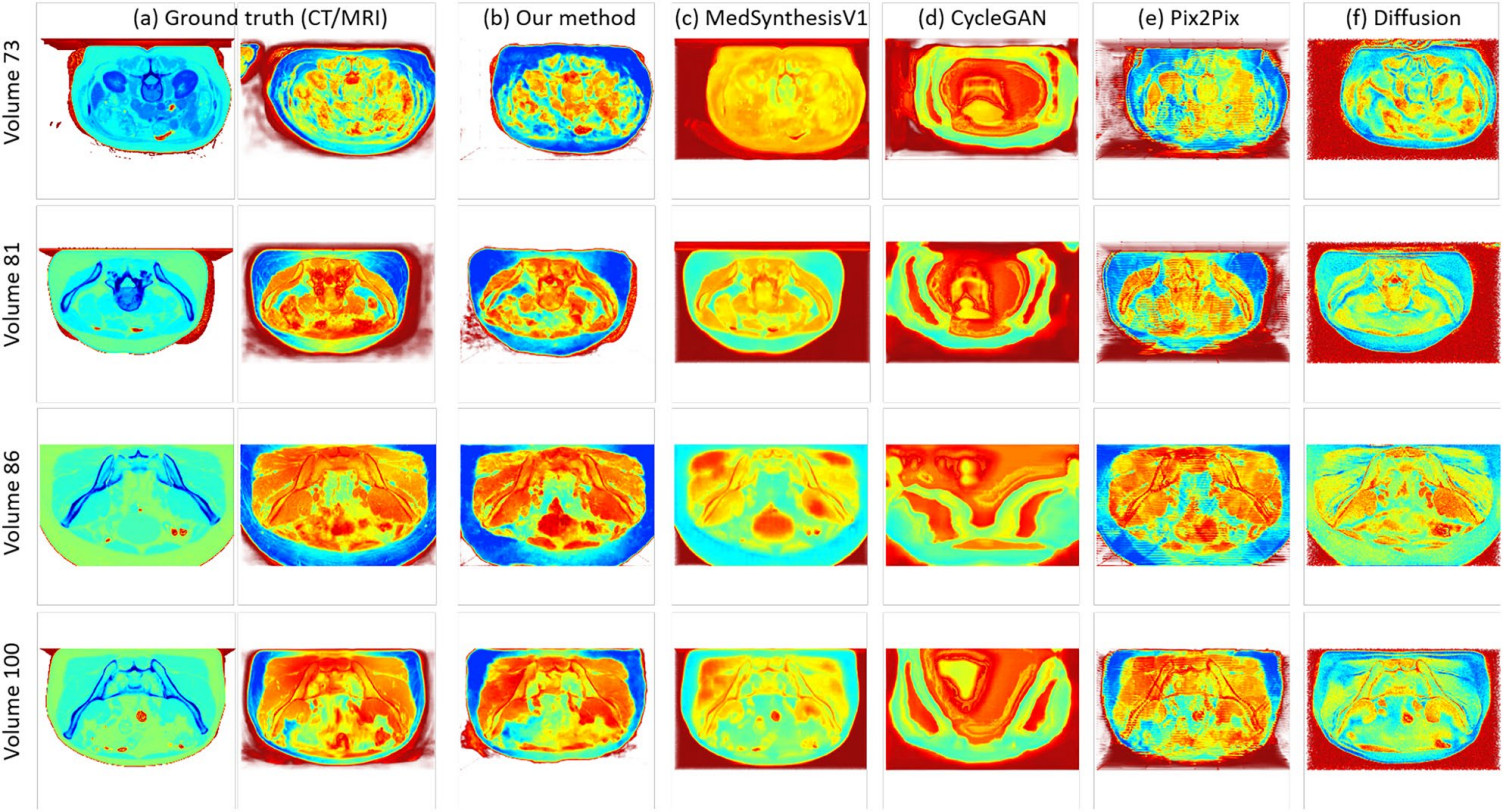
(**a**) volume visualization of the CT (left) and MRI (right) volumes from the ground truth, and (**b**–**f**) volume visualization of the synthesized MRI volumes from our method, MedSynthesisV1, CycleGAN, Pix2Pix, and Diffusion for the pelvis dataset. Each row denotes a specific volume with an associated number (the four volumes are randomly selected), and each column denotes a specific method used to generate those volumes.

**Fig. 7:**
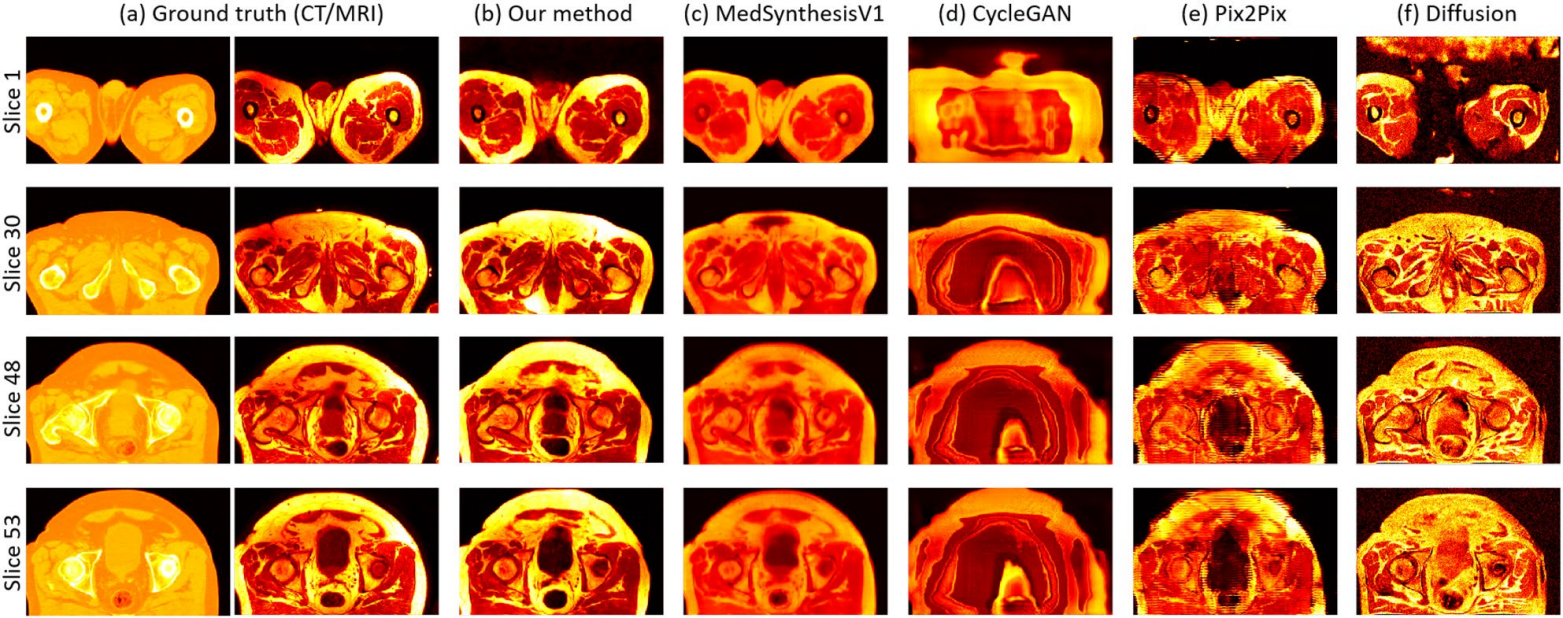
(**a**) slice visualization of the CT (left) and MRI (right) slices from the ground truth, and (**b**–**f**) slice visualization of the synthesized MRI slices from our method, MedSynthesisV1, CycleGAN, Pix2Pix, and Diffusion for the pelvis dataset. Each row denotes a specific slice with an associated number (the four slices are randomly selected from volume 85), and each column denotes a specific method used to generate those slices.

For the brain dataset, Fig. 4 displays the volume visualization results of inferred data from each method. Each row represents the results of one randomly selected volumetric dataset from the validation data (Each volumetric data is numbered, and we randomly select four volumes: 73, 87, 91, and 99). Each column represents the result of inferred data from each methods or CT/MRI ground truth after volume visualization. (a) shows the CT/MRI ground truth, (b) shows our method, (c) shows MedSynthesisV1, (d) shows CycleGAN, (e) shows pix2pix, and (f) shows Diffusion. Figure 5 shows the results of slice visualization of inferred data from each methods. Compared to the volume visualization results, each row in the slice visualization results shows one randomly selected slices from volume 71, specifically slices 60, 80, 90, and 100, and each column in the slice visualization results depicts the same content as in the volume visualization results. By examining these two figures, we can observe that compared to the MRI in the ground truth, our method produces the best results. MedSynthesisV1 also generates similar outer contours but fails to accurately capture the internal structures, with many internal features remaining blurry. CycleGAN produces the worst results, not only failing to present internal structures well but also showing a noticeable checkerboard effect. Pix2Pix’s results show an attempt to generate internal structures, but they exhibit errors in generating some brain cavity regions. Finally, Diffusion’s results show relatively good internal structures in the slice visualization, though there are some differences in contrast. However, in the volumetric visualization, Diffusion introduces some artifacts in the background area, which significantly degrades the quality of the results.

For the pelvis dataset, Fig. 6 displays the volume visualization results of inferred data from each methods, and Fig. 7 shows the results of slice visualization of inferred data from each methods. Each row and column in the figure represent the same information as in Figs. 4 and 5, but with different selected data. Here, we select volumes: 73, 81, 86, and 100, and slices: 1, 30, 48, and 53. From these two figures, we can observe that our method is the closest to the MRI in the ground truth, both in volume and slice visualizations. MedSynthesisV1 also generates relatively good results but exhibits errors in contrast and cannot produce particularly refined details. CycleGAN’s results are the poorest, as it can only generate an outline while performing poorly in other aspects. Pix2Pix also performs well but is somewhat blurry and has some horizontal artifacts. Diffusion also produces relatively good results but can exhibit varying contrast and may have some missing or extra parts. Therefore, the results from our method are the best.

### Evaluation metrics

Traditionally, PSNR/MSE and SSIM metrics are used to compare the image quality generated by different methods. However, Erfurt et al.^36^ pointed that PSNR/MSE does not always align with the human visual system (HVS) perception of image quality. Sometimes, the PSNR/MSE values between two nearly indistinguishable images can vary significantly; similarly, two images with the same PSNR/MSE may still exhibit noticeable differences in quality.

Therefore, we do not use PSNR/MSE to evaluate these methods. Instead, we select metrics that are more aligned with the human visual system. We select three metrics: MS-SSIM3^37^, FSIM^38^ and UQI^39^. MS-SSIM (Multi-Scale Structural Similarity) is an enhanced version of the structural similarity index that calculates image similarity across multiple scales. Unlike standard SSIM, MS-SSIM evaluates image quality by analyzing luminance, contrast, and structural information at various resolutions, providing a more comprehensive assessment of image similarity. FSIM (Feature Similarity Index) assesses the similarity between images by comparing their low-level features, such as edges and textures, which are more aligned with human visual perception. UQI (Universal Quality Index) is a metric designed to measure image quality by comparing the structural information between the reference image and the distorted image. Unlike some other metrics, UQI does not explicitly model human visual perception but provides a mathematical measure that aligns well with it. UQI is mathematically defined and does not explicitly employ a HVS model, but it surprisingly aligns well with HVS. For these metrics, higher values indicate greater similarity to the ground truth, while lower values indicate less similarity.

### Quantitative analysis

**Fig. 8:**
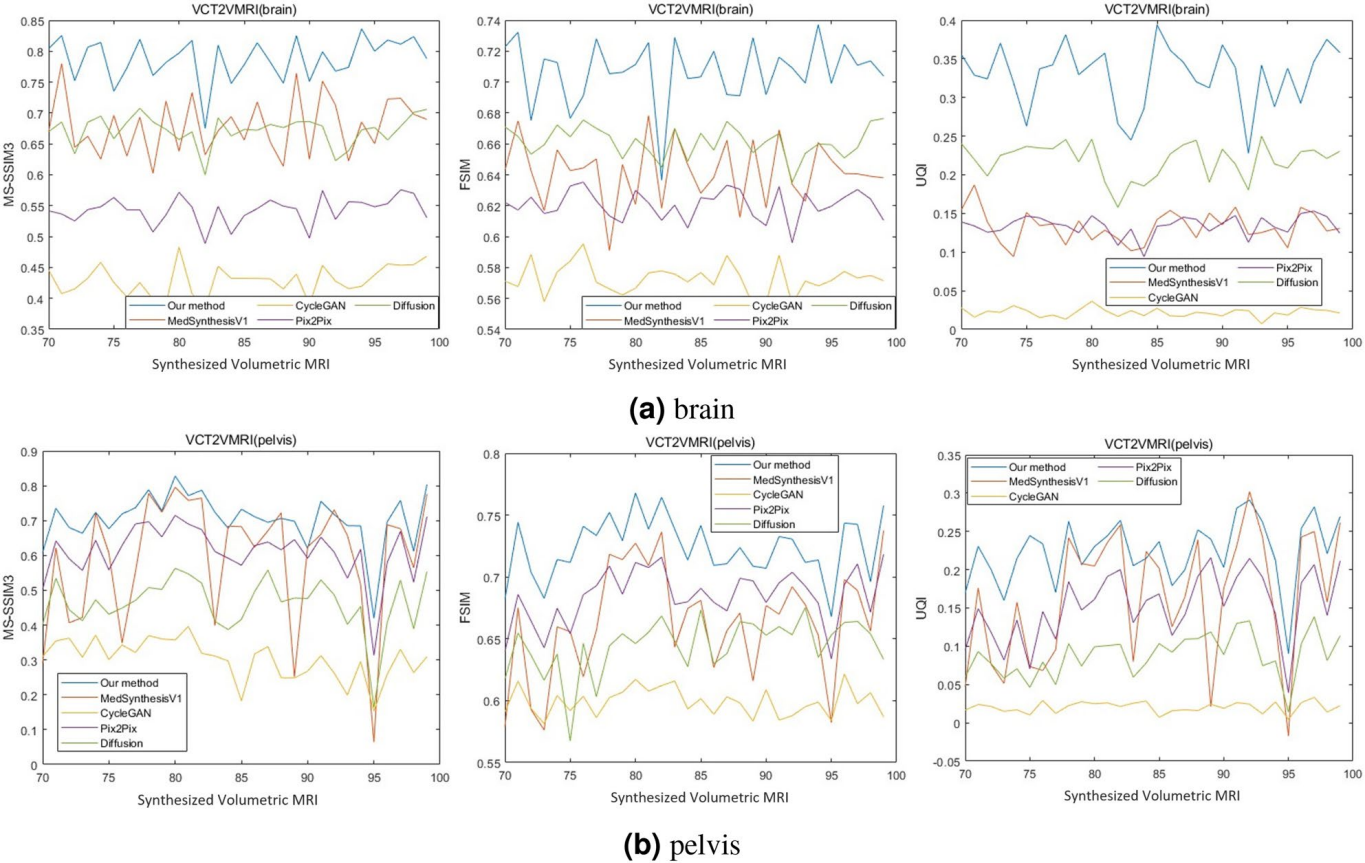
The MS-SSIM3, FSIM, UQI evaluation metrics of our method (blue), MedSynthesisV1 (orange), CycleGAN (yellow), Pix2Pix (purple), Diffusion model (green) for (**a**) brain and (**b**) pelvis datasets, respectively.

**Table 2:** The mean MS-SSIM3, FSIM, UQI evaluation metrics of our method, MedSynthesisV1, CycleGAN, Pix2Pix, Diffusion for brain and pelvis datasets (bold numbers indicate the best results).

Methods	Metrics					
	MS-SSIM3 (mean)		FSIM (mean)		UQI (mean)	
	Brain	Pelvis	Brain	Pelvis	Brain	Pelvis
Our method	**0.7879**	**0.7047**	**0.7070**	**0.7238**	**0.3285**	**0.2242**
MedSynthesisV1	0.6793	0.5927	0.6420	0.6640	0.1326	0.1643
CycleGAN	0.4296	0.3023	0.5727	0.5990	0.0218	0.0204
Pix2Pix	0.5423	0.6116	0.6205	0.6848	0.1342	0.1527
Diffusion	0.6723	0.4620	0.6615	0.6445	0.2188	0.0881

**Table 3:** Statistical comparison of Enhanced-DDPM with baseline models across multiple image quality metrics using paired t-tests for brain dataset.

Metric	Comparison	$M_{\text {diff}}$	*t*(df)	*p*	95% CI	*d*
MS-SSIM3	vs. MedSynthesisV1	0.109	*t*(29) = 1.411	$< 0.001$	[0.093, 0.124]	3.183
	vs. CycleGAN	0.358	*t*(29) = 6.338	$< 0.001$	[0.347, 0.370]	10.642
	vs. Pix2Pix	0.246	*t*(29) = 4.850	$< 0.001$	[0.235, 0.256]	7.261
	vs. Diffusion	0.116	*t*(29) = 2.276	$< 0.001$	[0.105, 0.126]	3.422
FSIM	vs. MedSynthesisV1	0.065	*t*(29) = 1.682	$<.001$	[0.057, 0.073]	3.222
	vs. CycleGAN	0.134	*t*(29) = 2.855	$<.001$	[0.125, 0.144]	6.615
	vs. Pix2Pix	0.086	*t*(29) = 2.060	$<.001$	[0.078, 0.095]	4.282
	vs. Diffusion	0.046	*t*(29) = 1.175	$<.001$	[0.038, 0.053]	2.260
UQI	vs. MedSynthesisV1	0.196	*t*(29) = 2.501	$<.001$	[0.180, 0.212]	5.033
	vs. CycleGAN	0.307	*t*(29) = 4.037	$<.001$	[0.291, 0.322]	7.538
	vs. Pix2Pix	0.194	*t*(29) = 2.827	$<.001$	[0.180, 0.208]	4.983
	vs. Diffusion	0.110	*t*(29) = 1.689	$<.001$	[0.096, 0.123]	2.846

Figure 8a shows the MS-SSIM, FSIM and UQI of the synthesized volume MRI from all methods, compared to the ground truth in the brain dataset. The horizontal axis represents the results of the synthesized volume MRI from different indexed original samples, while the vertical axis shows the MS-SSIM, FSIM and UQI. For the MS-SSIM3 metric, we observe from the leftmost graph that our method has the highest values for all synthesized volume MRI, exceeding the second-best method by approximately 0.1, whicih indicates that our method generates volume MRI that are most similar to the ground truth. MedSynthesisV1 and Diffusion have very similar MS-SSIM values, but some of MedSynthesisV1’s MS-SSIM values are slightly higher than those of Diffusion, making MedSynthesisV1 the second-best method and Diffusion the third-best. In contrast, CycleGAN has the lowest MS-SSIM values, significantly lower than those of other methods, making it the worst method. Pix2Pix’s MS-SSIM3 values fall between those of Diffusion and CycleGAN, making it the fourth-best method. For the FSIM metric, we observe from the middle graph that our method maintains the highest FSIM values for most of the synthesized volume MRI. Although Diffusion slightly surpasses our method for one volume MRI, the difference is not significant, so our method remains the best. Diffusion, Pix2Pix, and CycleGAN have decreasing FSIM values, each by approximately 0.5. MedSynthesisV1’s FSIM values are distributed between those of Diffusion and Pix2Pix, with some values exceeding Diffusion and some falling below Pix2Pix. Therefore, Diffusion, MedSynthesisV1, Pix2Pix, and CycleGAN rank second, third, fourth, and fifth, respectively. For the UQI metric, we observe from the rightmost graph that the order from top to bottom is our method, Diffusion, MedSynthesisV1/Pix2Pix, and CycleGAN. If MedSynthesisV1 and Pix2Pix are considered as a group, all the methods show a noticeable difference in UQI values, approximately around 0.1. Our method significantly exceeds the other methods, while MedSynthesisV1 and Pix2Pix have similar UQI values, indicating consistent image quality. And CycleGAN has extremely low UQI values. Therefore, our method is the best, followed by Diffusion, then MedSynthesisV1 and Pix2Pix, and finally, CycleGAN.

The Fig. 8bshows the MS-SSIM3, FSIM, and UQI of the synthesized volume MRI from all methods in reference to the ground truth in the plevis dataset. From the figure, we can see that the MS-SSIM3, FSIM, and UQI, from highest to lowest, all are our method, Pix2Pix, Diffusion, and CycleGAN. The values of MedSynthesisV1 lie between our method and CycleGAN, sometimes even exceeding our method and at other times falling below CycleGAN, indicating instability. Therefore, in the pelvis dataset, our method is the best, CycleGAN is the worst, and the other methods fall in between.

Additionally, the Table 2 shows the average MS-SSIM3, FSIM and UQI of the synthesized volume MRI from each method compared to the ground truth in both the brain and pelvis datasets. In the table, we can see that our method has the highest average values in both datasets, indicating that our synthesized results are the closest to the ground truth. In contrast, CycleGAN has the lowest average values, indicating that its synthesized results are the least similar to the ground truth. And in the brain dataset, MedSynthesisV1 has a higher MS-SSIM3 than Diffusion, which is higher than Pix2Pix. However, Diffusion has higher FSIM values than MedSynthesisV1, which are higher than Pix2Pix, Diffusion’s UQI values are higher than Pix2Pix, which are higher than MedSynthesisV1 . In the pelvis dataset, Diffusion’s MS-SSIM3 and FSIM values are higher than MedSynthesisV1, which are higher than Pix2Pix, while MedSynthesisV1’s UQI values is higher than Diffusion, which is higher than Pix2Pix. Therefore, the relative performance of MedSynthesisV1, Diffusion, and Pix2Pix is difficult to determine definitively.

To rigorously assess the superiority of our Enhanced-DDPM, we conducted paired *t*-tests comparing its performance against all baseline models across three key image quality metrics: MS-SSIM, FSIM, and UQI. The detailed statistical results are summarized in Table 3. The analysis reveals that Enhanced-DDPM achieved statistically significant improvements over every baseline model on all metrics (all $p <.001$). The magnitude of improvement, as indicated by the mean differences ($M_{\text {diff}}$), was substantial. Notably, the most pronounced advantage was observed against CycleGAN in the MS-SSIM metric ($M_{\text {diff}} = 0.358$, $t(29) = 6.338$, $p <.001$), representing a very large effect size. Consistent and significant advantages were also evident against Pix2Pix and MedSynthesisV1 across all measures. Even compared to the standard Diffusion model baseline, our enhanced architecture delivered significant gains (e.g., for MS-SSIM: $M_{\text {diff}} = 0.116$, $t(29) = 2.276$, $p <.001$).These results provide compelling statistical evidence that the architectural innovations in Enhanced-DDPM—namely the Transform Block (TB) and Self-attention Less Memory Block (SALMB)—translate into a quantitatively superior and robust ability for volumetric CT-to-MRI synthesis, outperforming existing GAN-based, CNN-based, and diffusion-based approaches.

### Performance and complexity analysis

**Table 4:** The performance of our method, including both training time and inference time consumed by our enhanced-ddpm, and the time consumed by global normalization and automatic contrast enhancement, for brain and pelvis datasets.

Dataset	Number of epochs	Training time (h)	Inference time (h)	Global normalization and automatic contrast enhancement (s)
Brain	4250	102.49	17.40	17.19
Pelvis	5000	134.76	25.45	17.43

Table 4 shows the number of epochs, training and inference time, and time of global normalization & automatic contrast enhancement for our method on the brain and pelvis datasets. Due to the Diffusion-based nature of our method, the inference time is relatively long. However, the global normalization and automatic contrast enhancement requires very little time but significantly improves the results, demonstrating the effectiveness of global normalization and automatic contrast enhancement.

**Table 5:** The floating point operations per second (FLOPs), total number of parameters, and memory consumed for the brain and pelvis datasets.

Dataset	FLOPs (Giga)	Total number of parameters	Memory consumed (MB)
Brain	1195.46	11,12,64,513	7467
Pelvis	1793.58	11,12,64,513	9389

Table 5 reports the floating point operations per second (FLOPs), total number of parameters, and GPU memory consumed for both brain and pelvis datasets.

### Statics of imperfectly-paired data

**Table 6:** The statistics of both imperfectly-paired slices and volumes involved in the brain and pelvis datasets.

Statistics	Brain	Pelvis
Total paired slices	16,000	9600
# of imperfectly-paired slices	832	531
Ratio of imperfectly-paired slices	5.2%	5.53%
Total paired volumes	100	100
# of imperfectly-paired volumes	46	24
Ratio of imperfectly-paired volumes	46%	24%

Due to the fact that paired volume CT and MRI collected from different modalities at different times often contain a certain amount of imperfectly paired slices, the task of volume CT-to-MRI translation is more challenging compared to image-based CT-to-MRI translation. And Table 6 presents the statistics of imperfectly paired slices and volumes in the brain and pelvis datasets, which were calculated using a specially developed automatic program. A slice is considered imperfectly paired if the foreground area difference exceeds 15% of the total slice area. A volume is deemed imperfectly paired if it contains any imperfectly paired slices. According to Table 6, there are 832 and 531 imperfectly paired slices in the brain and pelvis datasets, respectively, accounting for 5.2% and 5.53% of the total paired slices. There are 46 and 24 imperfectly paired volumes for the two datasets, respectively, accounting for 46% and 24% of the total paired volumes.

### Parameter selection

**Fig. 9:**
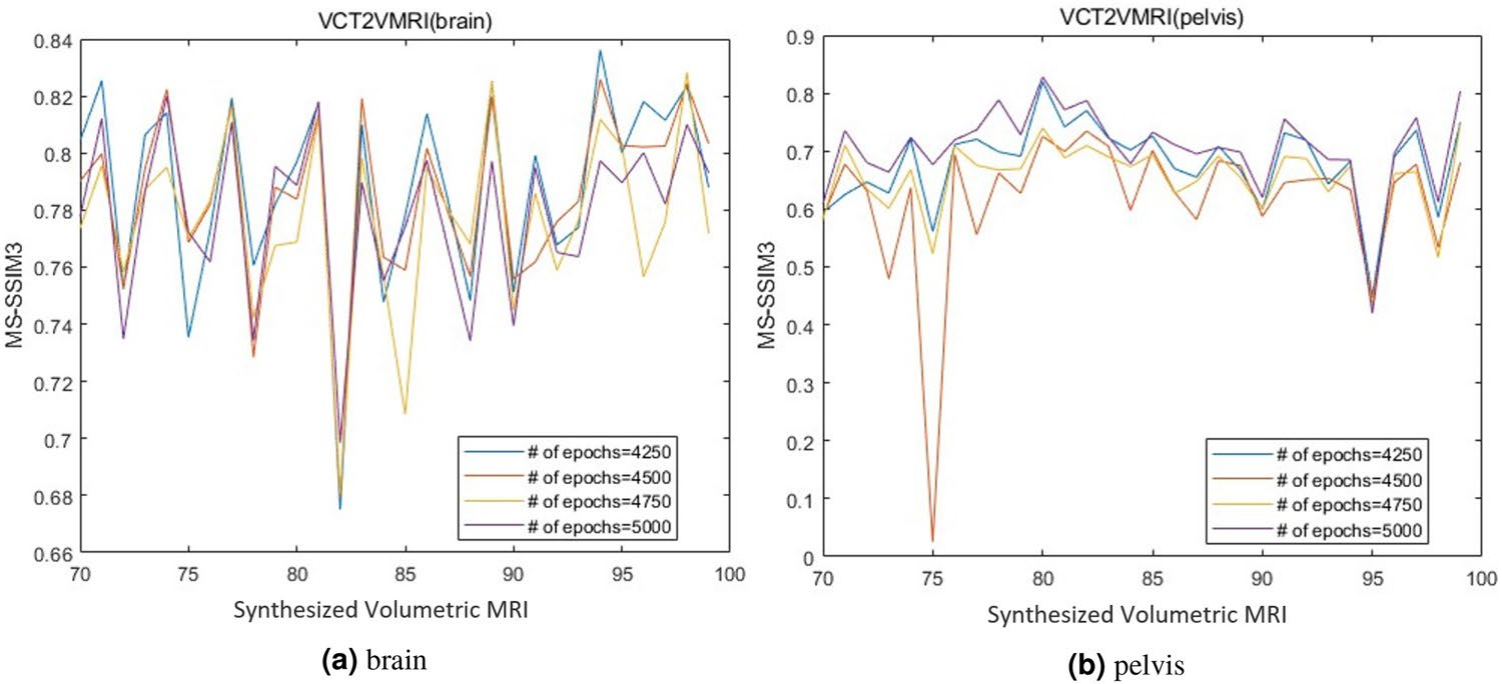
Selection of number of epochs for (**a**) brain and (**b**) pelvis datasets, respectively.

**Fig. 10:**
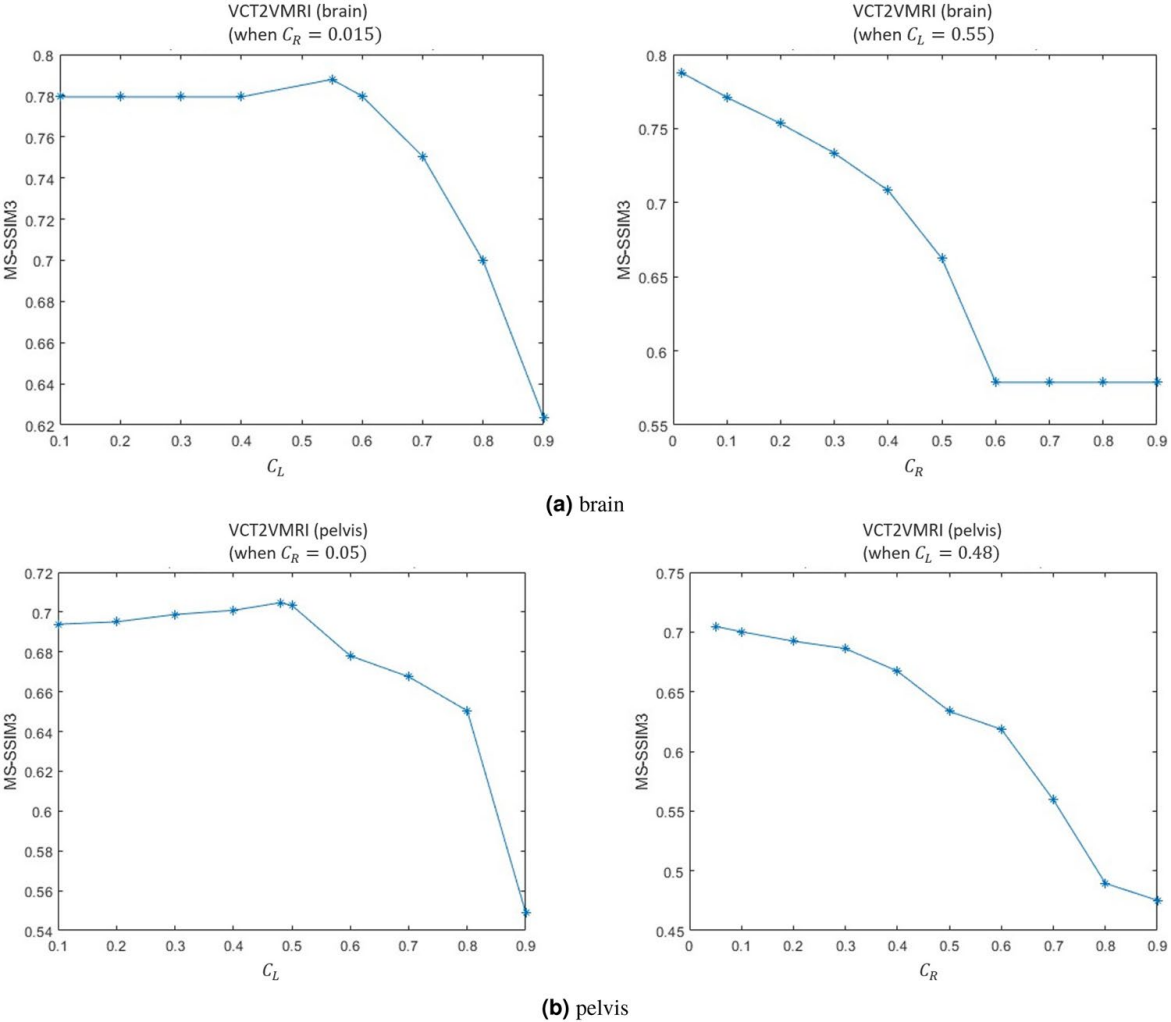
Selection of parameters of and for (**a**) brain and (**b**) pelvis datasets, respectively.

Our method has two key hyperparameters to determine: the number of training epochs for inference and the parameters $cutoffPercentage\_left$ and $cutoffPercentage\_right$ mentioned in Algorithm 1. We determine the optimal number of training epochs by evaluating the MS-SSIM3 values for inference results at different epoch counts. Figure 9 shows the MS-SSIM3 for various epoch numbers on the brain and pelvis datasets. For the brain dataset, it is challenging to distinguish the best and worst SSIM values among different epoch numbers due to their overlap. Generally, we find that 4250 epochs yield the best MS-SSIM3 values across different samples, so this number is chosen for inference. For the pelvis dataset, MS-SSIM3 values are highest at 5000 epochs, so 5000 is selected as the number of epochs for inference.

The Fig. 10 display the MS-SSIM3 of our method’s synthesized volume data under different $cutoffPercentage\_left$ and $cutoffPercentage\_right$. To control for the impact of one parameter on the other, we fixed $cutoffPercentage\_left$ and $cutoffPercentage\_right$ at 0.015 and 0.55 during the brain dataset experiments, and at 0.05 and 0.48 during the pelvis dataset experiments. For the brain dataset, in the left figure, we can see that the MS-SSIM3 values remain almost unchanged when the $cutoffPercentage\_left$ is less than or equal to 0.4. Then it rises and reaches its peak around 0.55, after which the MS-SSIM3 values continuously decrease. Therefore, our method achieves the best synthesis result when $cutoffPercentage\_left$ is 0.55. In the right figure, when $cutoffPercentage\_right$ is 0.015, the MS-SSIM3 values are highest, and the MS-SSIM3 values continuously decreases when the $cutoffPercentage\_right$ is less than or equal to 0.6, and then levels off. Thus, we choose 0.015 as the optimal $cutoffPercentage\_right$. For the pelvis dataset, in the left figure, we can see that the MS-SSIM3 values continuously increase when the $cutoffPercentage\_left$ is less than or equal to 0.48. Beyond 0.48, the MS-SSIM3 values start to decrease, so we choose 0.48 as the optimal $cutoffPercentage\_left$ value. In the right figure, when $cutoffPercentage\_left$ is 0.05, the MS-SSIM3 values are highest, and the MS-SSIM3 values continuously decrease as the $cutoffPercentage\_right$ increases. Therefore, we choose 0.05 as the optimal $cutoffPercentage\_right$ value.

### Limitations

While our proposed method shows promising results in generating MRI images from CT scans, it is important to acknowledge the limitations inherent in generative models, particularly in clinical contexts. One known limitation is the occurrence of “hallucinations” or artifacts in the generated images. These artifacts may not accurately represent the real clinical scenarios and could potentially interfere with clinical decision-making. In critical applications, such as tumor detection and treatment planning, these inaccuracies could lead to suboptimal outcomes.

Dependence on MRI Acquisition Protocol. A significant limitation of our current study is its reliance on MRI data from a single, specific acquisition protocol (T1-weighted). As noted by the reviewer, clinical MRI encompasses a wide variety of sequences (e.g., T2-weighted, FLAIR, DWI) and parameters that profoundly affect image appearance and quantitative values. Our model, trained exclusively on T1w images, is inherently biased towards generating synthetic MRIs with T1w-like contrast and texture. Its performance may substantially decrease when applied to CT scans intended to synthesize MRI images from a different contrast mechanism (e.g., converting CT to synthetic T2w images). This protocol dependence limits the immediate broad applicability of the model in clinical settings where multiple and variable sequences are routine.

Clinical Translation and Diagnostic Caveats. While this work demonstrates technical feasibility, its path to clinical application requires careful consideration of unique risks compared to the more established inverse task of MRI-to-CT synthesis. Successful clinical adoption of synthetic medical images has primarily been in contexts where the synthetic image is not used for primary diagnosis, such as generating CT scans for PET attenuation correction or radiotherapy dose calculation. In these cases, the key requirement is accurate electron density mapping, and minor inaccuracies in anatomical detail may have limited clinical impact. In stark contrast, the synthesis of diagnostically rich MRI from CT introduces higher stakes. A critical limitation is the potential for the model to hallucinate or misrepresent anatomical or pathological features. For instance, inaccurately mapping a CT artifact or a region of normal tissue variation into a synthetic MRI signal mimicking a tumor could lead to severe diagnostic errors. This inherent risk contributes to the relative scarcity of studies on CT-to-MRI mapping compared to the opposite direction. Therefore, we emphatically caution that our current model is a proof-of-concept and should not be used for direct diagnostic purposes. Its potential utility lies rather in scenarios where MRI is inaccessible (e.g., emergency assessment, resource-limited settings, or patients with MRI contraindications), serving as a supplementary visual aid for planning or triage. Any clinical use must involve cautious interpretation by experts alongside the original CT and full clinical context. Future work must incorporate robust uncertainty quantification and rigorous clinical validation to mitigate these risks before any diagnostic application can be considered.

## Conclusions and future works

Traditionally, obtaining Medical inter-modality imaging results requires using various medical devices to scan patients, which can be time-consuming, costly, and potentially harmful to the patient. Therefore, we propose using deep learning to achieve CT-to-MRI inter-modality translation. In addition, due to the presence of volumetric data in medical imaging results, it is necessary to achieve volume-based CT-to-MRI inter-modality translation, which is more challenging compared to image-based CT-to-MRI inter-modality translation. To address this problem, we propose a method based on a novel Diffusion model named enhanced-DDPM and incorporating a novel post-processing algorithm called global normalization and automatic contrast enhancement. Our method can robustly synthesize volume MRI from volume CT, even in the presence of approximately 6% of imperfectly paired slices.

To validate our method, we conduct experiments on brain and pelvic datasets obtained from clinical practice. We also compare our method with state-of-the-art techniques, both qualitatively and quantitatively. Our experimental results show that, our method outperforms state-of-the-art techniques, including MedSynthesisV1 (11%, 7%, 20% better in average MS-SSIM3, average FSIM, average UQI for brain; 12%, 6%, 6% better in average MS-SSIM3, average FSIM, average UQI for pelvis), CycleGAN (36%, 13%, 31% better in average MS-SSIM3, average FSIM, average UQI for brain; 40%, 12%, 20% better in average MS-SSIM3, average FSIM, average UQI for pelvis), Pix2Pix (25%, 9%, 19% better in average MS-SSIM3, average FSIM, average UQI for brain; 9%, 4%, 7% better in average MS-SSIM3, average FSIM, average UQI for pelvis) and Diffusion(12%, 5%, 11% better in average MS-SSIM3, average FSIM, average UQI for brain; 25%, 8%, 14% better in average MS-SSIM3, average FSIM, average UQI for pelvis) when using ground truth as a reference. Finally, we conduct an experiment to select the optimal hyperparameters, including the number of epochs, the $cutoffPercentage\_left$ and $cutoffPercentage\_right$. The experimental results indicate that the optimal number of epochs is 4,250 for brain dataset and 5,000 for pelvis dataset. The optimal $cutoffPercentage\_left$ is 0.55 for brain dataset and 0.48 for pelvis dataset, while the $cutoffPercentage\_right$ for the right side is 0.55 for brain and 0.05 for pelvis dataset.

There are five future directions we expect to do. First, introduce more types of modalities (such as text, images, and audio) to enhance the model’s generalization capability. Simultaneously, increase the diversity of datasets to ensure that the model remains robust across different scenarios. Second, since we are using Diffusion models, which typically require a longer inference time, we can consider using methods like DDIM to accelerate the inference process. Third, generative models can sometimes produce artifacts or “hallucinations” that may not accurately reflect real clinical scenarios. To mitigate these issues, future work will focus on improving the quality and reliability of generated images, possibly by incorporating advanced regularization techniques or exploring post-processing methods to remove artifacts. Additionally, further validation on larger, more diverse datasets could help assess the generalizability of the model and its robustness in real-world clinical settings. Fourth, collaborate with clinicians to conduct user studies and evaluate how synthesized MRI images affect diagnosis, treatment decisions, and patient outcomes. Fifth, addressing Dependence on MRI Acquisition Protocols. Develop conditioned diffusion models that incorporate the target MRI contrast (e.g., T1w vs. T2w) as an additional input to the network; explore disentanglement techniques to separate anatomical content from sequence-specific styles; and train on large, multi-contrast, multi-center datasets to enhance the model’s robustness and generalizability across different acquisition protocols.

## Data Availability

The data that support the findings of this study are openly available in Grand Challenge repository at https://synthrad2023.grand-challenge.org/.
